# Ruxolitinib for myelofibrosis

**DOI:** 10.3892/etm.2013.886

**Published:** 2013-01-07

**Authors:** LIAN GU, LI SU, QING CHEN, JUANJUAN XIE, GUANGLIANG WU, YAN YAN, BAOYUN LIANG, JINJING TAN, NONG TANG

**Affiliations:** 1 Department of Internal Neurology, First Affiliated Hospital, Guangxi University of Chinese Medicine; Nanning, Guangxi, P.R. China; 2 School of Public Health of Guangxi Medical University; Nanning, Guangxi, P.R. China; 3 Principal’s Office, Guangxi University of Chinese Medicine, Nanning, Guangxi, P.R. China

**Keywords:** ruxolitinib, myelofibrosis, randomised controlled trial

## Abstract

The aim of the present study was to assess the beneficial and harmful effects of ruxolitinib in patients with myelofibrosis (MF). The Cochrane databases, PubMed and Embase were searched for studies published up to October 2012. Randomised controlled trials assessing ruxolitinib versus a placebo or the best available therapy in patients with MF were included. Two trials randomised 528 patients with MF to ruxolitinib versus a placebo or ruxolitinib versus the best available therapy. Compared with the placebo, ruxolitinib had a significant beneficial effect on the proportion of patients that had a reduction in spleen volume of ≥35% at 24 weeks [odds ratio (OR), 109.78; 95% confidence interval (CI), 14.97–804.78] or an increased overall survival rate (OR, 2.02; 95% CI, 0.99–4.12). Ruxolitinib significantly increased the risk of several non-haematological or haematological adverse events, but not the risk of treatment discontinuations (OR, 1.04; 95% CI, 0.50–2.14). Compared with the best available therapy, ruxolitinib had a significant beneficial effect on the proportion of patients that had a reduction in spleen volume of ≥35% at 24 (OR, 68.45; 95% CI, 4.15–1129.19) or 48 weeks (OR, 56.20; 95%CI, 3.40–928.67). Ruxolitinib once again significantly increased the risk of several non-haematological adverse events, serious adverse events and dose reductions or interruptions (OR, 9.60; 95% CI, 4.66–19.81), but not the risk of treatment discontinuations (OR, 1.54; 95% CI, 0.48–4.97). In conclusion, based on the trials included in the present study, the use of ruxolitinib is beneficial in the treatment of MF.

## Introduction

Myelofibrosis (MF) is a rare disorder of the bone marrow which is characterised by excessive production of reticulin and collagen fibers (1). MF includes primary MF (PMF), post-essential thrombocythaemia (post-ET) MF and post-polycythaemia vera (post-PV) MF (2). Among the Philadelphia chromosome-negative chronic myeloproliferative neoplasms (MPNs), MF is the most symptomatic and carries the worst prognosis (3). According to epidemiological studies, it is estimated that the incidence of MF may be as high as 1.5 per 100,000 individuals. In clinical series, the median age at diagnosis for the majority of patients was ∼65 years. In total, ∼22% of the cases occur in patients younger than 56 years old and ∼11% in patients younger than 46 years old (2). Other studies (4–8) indicate that 10–15% of cases may transform into secondary MF by the end of the second decade subsequent to PV or ET diagnosis. In 2010, MF continued to be the MPN that caused the highest morbidity and was corelated with the poorest life expectancy (9). The most common abnormalities in patients with MF include progressive splenomegaly, cytopenias and bothersome constitutional symptoms (e.g., unintentional weight loss, fevers and debilitating fatigue) (10). MF is also able to induce blastic transformation (11) and mortality (12).

Numerous conventional therapeutic modalities have been used in MF treatment as additional supportive treatments (13). Until recently, allogeneic haematopoietic stem cell transplantation has been the only potentially curative treatment for patients with MF, but an option that was traditionally only feasible for a small percentage of patients, namely the younger and physically fit. However, recent studies suggest that it may be utilised in older individuals as well (14,15). Other therapeutic modalities (e.g., thalidomide, hydroxyurea, corticosteroids, anagrelide, androgens, splenectomy or spleen irradiation, transfusions, pirfenidone and suramin) are only palliative and do not provide a substantial improvement in survival rates (16–32).

Ruxolitinib (formerly known as INC424 or INCB18424) is an orally bioavailable, selective Janus kinase (JAK) 1 and 2 inhibitor approved by the US Food and Drug Administration for the treatment of MF. The mechanism of action of ruxolitinib is the attenuation of cytokine signalling by the inhibition of JAK1 and JAK2 (wild-type or mutated forms), leading to antiproliferative and proapoptotic effects. On the basis of the results of a phase II clinical trial in patients with MF, ruxolitinib showed durable efficacy in the reduction of splenomegaly and circulating pro-inflammatory cytokines. Ruxolitinib recipients also exhibited improvements in physical activity, weight gain, existing symptoms (including constitutional symptoms) and parameters gauging quality of life. These findings were validated by two phase III clinical MF studies (1,33,34).

Conventional medications for treating MF are largely palliative and rarely provide durable clinical benefits, whereas stem cell transplantation appears to be restricted to a minority of patients. Given the limitations already discussed, more effective disease-targeted therapeutic approaches are required for patients with MF. Based on the efficacy and tolerability reported in completed clinical trials, ruxolitinib now has an important place among currently available treatment modalities. In the present systematic review, the beneficial and harmful effects of ruxolitinib in patients with MF are assessed.

## Materials and methods

### Data sources

The Cochrane Central Register of Controlled Trials in The Cochrane Library, PubMed and Embase were searched for studies published up to October 2012 using the search terms ‘myelofibrosis’, ‘ruxolitinib’ and ‘INCB-018424’.

### Study selection

Randomised controlled trials assessing ruxolitinib versus no intervention, a placebo or the best available therapy in patients with intermediate risk, high risk or not determined MF, including PMF, post-PV MF and post-ET MF, were included, irrespective of gender, age or ethnic origin.

### Outcome measures

The primary outcome measures were: i) the proportion of patients that had a reduction in spleen volume of ≥35% at 24 weeks; ii) the proportion of patients that had a reduction in spleen volume of ≥35% at 48 weeks. The secondary outcome measures were: i) overall survival rate; ii) all adverse events, including non-haematological and haematological adverse events, serious adverse events, necessary dose reductions or interruptions and treatment discontinuations.

### Assessment of trial methodological quality

The methodological quality of each trial included in the review was assessed by two authors using the quality criteria specified in the Cochrane Handbook for Systematic Reviews of Interventions 5.1.0 (35). The assessment was based on adequate sequence generation, allocation concealment, blinding, selective outcome reporting and other sources of bias. Any disagreements were resolved through consensus. Each criteria was classified as ‘yes’, ‘no’ or ‘unclear’. A classification of ‘yes’ indicated the above assessment methods had been used and were specifically described, ‘no’ if it did not mention the above assessment methods, and ‘unclear’ if it only mentioned the above assessment methods had been used but did not specifically describe them. The summary assessments for the bias risk for each significant outcome within and across studies were classified as ‘low risk of bias’, ‘high risk of bias’ and ‘unclear risk of bias’.

### Data extraction

Data were extracted independently by two authors in a standard format. Any disagreements were resolved through consensus. Authors of the eligible studies were contacted to request missing data that were required for further analysis. The primary author, country, study design, sample size, age range, number of males and females, initial dose, intervention, median follow-up, methodological quality of the trial and the outcome measures were extracted from each trial as described.

### Data synthesis

Data synthesis and analysis was performed following the recommendations of Cochrane Review Manager software, RevMan version 5.1.0. All data were analysed on the basis of an intention to treat and therefore included all patients irrespective of compliance or follow-up. The random-effects (36) and fixed-effects models (37) were used for analysing the data. If the models provided the same result with regard to statistical significance, only the results of the fixed-effect model were presented. Odds ratios (ORs) with 95% confidence intervals (CIs) were used for the analysis of all outcomes. Heterogeneity was explored by the Chi-square test, with the significance set at a P-value of 0.10 and measured by I^2^(38). Possible sources of heterogeneity were assessed by sensitivity and subgroup analyses as described later. Funnel plots and other analytical methods were used to assess potential bias, depending on the number of clinical trials included (39).

### Subgroup analyses and sensitivity analysis

In order to explore the effect of size differences, the present study aimed to perform analyses on the following subgroups: i) MF type (PMF, post-PV MF or post-ET MF); ii) IPSS risk group (intermediate-2, high or not determined); iii) age (≤65 or >65 years); iv) JAK2V617F mutation status (presence or absence); v) baseline palpable spleen length (≤10 or >10 cm); vi) baseline haemoglobin level (≥10 or <10 g/dl); vii) gender. Sensitivity analyses were performed to exclude the trials which would potentially bias the results.

## Results

### Search results

A total of 307 references were identified through electronic searches of the Cochrane Central Register of Controlled Trials in the Cochrane Library (n=2), PubMed (n=8) and Embase (n=223). Duplicates, non-clinical studies, clearly irrelevant articles or studies with a different purpose (n=303) were excluded. There were no quasi-randomised studies identified. A total of 4 references were retrieved for further assessment and another 2 non-randomised control trials were excluded. All included trials had been published as full manuscripts.

### Characteristics of included studies

Together the two trials included 528 patients and used a parallel group design. The first trial, conducted by Verstovsek *et al*(33)*,* used a placebo control, while the second trial, conducted by Harrison *et al*(34), used the best available therapy as a control. The first trial was conducted at 89 sites in the United States, Australia and Canada and patients were randomised (1:1) to receive ruxolitinib or a placebo. Patients of the second trial were randomised (2:1) to ruxolitinib or to the best available therapy. The mean age (range, 35–91 years) of the patients included and the proportion of males (55.3%) was reported in each trial. The median spleen volume was >2,300 cm^3^ (>10 times the median normal spleen volume of 200 cm^3^). A total of 38.8% of the patients had an IPSS intermediate-2-risk disease, 60.61% had a high-risk disease and 0.1% were not determined. A total of 51.1% of the patients were categorized as PMF, 31.3% as post PV MF and 17.6% as post ET MF. The proportion of patients who underwent previous MF therapy with hydroxyurea and radiotherapy was 66.48 and 1.82%, respectively. On the basis of the baseline peripheral blood platelet count (Plt), in the first trial (33) the initial dose of ruxolitinib was 15 mg/bid (Plt, 100–200x10^9^/l) or 20 mg/bid (Plt, >200×10^9^/l). In the second trial (34) the initial dose of ruxolitinib was also 15 or 20 mg/bid and was adjusted to within the range of 5 to 25 mg/bid.

### Risk of bias in included studies

Each study only described a randomisation but did not describe the method used for generating and concealing the allocations, so were consequently assessed as ‘unclear’. The first trial (33) was described as double-blind and placebo-controlled. Although there was no information with regard to which aspects involved were blinded, it was assessed as ‘yes’. The second trial (34) had no information on blinding, therefore it was uncertain whether blinding was used or not and consequently an assessment of ‘unclear’ was made. An intention-to-treat analysis was used in the two trials and each were assessed as ‘yes’. All outcomes of the trials listed in their methods sections were reported in their results, therefore this criteria was also assessed as ‘yes’. The first trial (33) was supported by Incyte and the second trial (34) was supported by Novartis pharmaceuticals, so each trial was assessed as ‘no’.

### Effects of interventions

It was not possible to perform meta-analysis as the two studies included had different comparison interventions and were not groupable.

### Ruxolitinib versus placebo

In the trial conducted by Verstovsek *et al*(33), when compared with the placebo, ruxolitinib significantly increased the proportion of patients that had a reduction in spleen volume of ≥35% at 24 weeks (OR, 109.7; 95% CI, 14.97–804.78). Ruxolitinib also had a significant beneficial effect on overall survival rate (OR, 2.02; 95% CI, 0.99–4.12). There were no significant differences identified in the treatment discontinuations between ruxolitinib and the placebo (OR, 1.04; 95% CI, 0.50–2.14). The most frequently reported grade 1 or 2 non-haematological and haematological adverse effects in the ruxolitinib group were fatigue and anaemia, respectively. The most frequently reported grade 3 or 4 non-haematological adverse effects in the ruxolitinib group were fatigue, abdominal pain, diarrhoea and arthralgia, while anaemia and thrombocytopenia were the most common haematological adverse effects. No data were reported on the proportion of patients that had a reduction in spleen volume of ≥35% at 48 weeks, serious adverse effects or adverse effects of any grade requiring dose reductions or interruptions (Table I).

### Ruxolitinib versus best available therapy

In the trial conducted by Harrison *et al*(34), when compared with the best available therapy, ruxolitinib significantly increased the proportion of patients that had a reduction in spleen volume of ≥35% at 24 weeks (OR, 68.45; 95% CI, 4.15–1129.19). Ruxolitinib also significantly increased the proportion of patients that had a reduction in spleen volume of ≥35% at 48 weeks (OR, 56.20; 95% CI, 3.40–928.67). There were no significant differences in the treatment discontinuations between ruxolitinib and the best available therapy (OR, 1.54; 95% CI, 0.48–4.97), however ruxolitinib significantly increased all grades of adverse effects that required dose reductions or interruptions (OR, 9.60; 95% CI, 4.66–19.81). In the ruxolitinib group, the most common serious adverse effect was anaemia. Other commonly reported serious adverse events included abdominal pain, pyrexia, esophageal varices, dyspnea and pneumonia. The non-haematological adverse event that occurred in any grade more frequently in the ruxolitinib group was diarrhoea [with diarrhoea of any grade occurring in 34 of 146 patients (23%) and grade 3 or 4 diarrhoea occurring in 2 of 146 patients (1%)]. The most common grade 3 or 4 non-haematological adverse event occurring in the ruxolitinib group was abdominal pain (occurring in 3% of the patients). Other commonly reported grade 3 or 4 non-haematological adverse events included abdominal pain, back pain and pyrexia. No data were reported on overall survival rates (Table I).

### Subgroup and sensitivity analyses

Subgroup or sensitivity analyses were not conducted in the present study due to the lack of sufficient trial numbers.

## Discussion

In total, the two trials randomised 528 patients with MF to ruxolitinib versus a placebo or ruxolitinib versus the best available therapy. Compared with the placebo, the present study identified that ruxolitinib had a significant beneficial effect on the proportion of patients that had a reduction in spleen volume of ≥35% at 24 weeks and an increased overall survival rate. Ruxolitinib significantly increased the risk of several non-haematological or haematological adverse events, but not the risk of treatment discontinuations. Compared with the best available therapy, ruxolitinib had a significant beneficial effect on the proportion of patients that had a reduction in spleen volume of ≥35% at 24 or 48 weeks. Ruxolitinib significantly increased the risk of several non-haematological adverse events, serious adverse events and dose reductions or interruptions, but not the risk of treatment discontinuations.

In the two randomised controlled trials on ruxolitinib in MF, the method to generate randomisation sequences was not stated, concealment of randomisation was not mentioned, information on blinding was not available in one study, the total number of included patients was small, follow-up was insufficient and no data were obtained on changes in marrow fibrosis or the JAK2V617F allele burden. A potential bias existed due to the limitations of the search strategy in the present study. Owing to insufficient experience among the authors, a second potential bias involved the assessment of the methodological quality of the trials included.

Based on the two trials, there is evidence to support the use of ruxolitinib for the treatment of MF. However, ruxolitinib should be used under close supervision from a physician as it is associated with the risks of potentially serious adverse effects, including anaemia and pyrexia. High-quality randomised controlled trials with longer follow-ups are required to reach firm conclusions with regard to the ability of ruxolitinib to modify the natural disease course.

## Figures and Tables

**Table I.** Comparison of the proportion of patients that had a reduction in spleen volume of ≥35% at 24 or 48 weeks, overall survival rate, discontinued treatment owing to adverse effects and adverse effects of any grade requiring dose reductions or interruptions, between the trial intervention (ruxolitinib) and the trial control (a placebo or the best available therapy).

	Reduced spleen volume of ≥35% at 24 weeks				Reduced spleen volume of ≥35% at 48 weeks				Overall survival rate			
First author, year	Intervention (events/total)	Control (events/total)	Statistical method	Effect estimate	Intervention (events/total)	Control (events/total)	Statistical method	Effect estimate	Intervention (events/total)	Control (events/total)	Statistical method	Effect estimate
Verstovsek *et al,* 2012	65/155	1/153	OR (M–H, Fixed, 95% CI)	109.78 (14.97, 804,78)	-	-	-	-	142/155	130/154	OR (M–H, Random, 95% CI)	2.02 (0.99, 4.12)
Harrison *et al,* 2012	46/144	0/72	OR (M–H, Fixed, 95% CI)	68.45 (4.15, 1129.19)	40/144	0/72	OR (M–H, Fixed, 95% CI)	56.20 (3.40, 928.67)	-	-	-	-

	Discontinued treatment due to adverse effects				Adverse effects requiring dose reductions or interruptions			
First author, year	Intervention (events/total)	Control (events/total)	Statistical method	Effect estimate	Intervention (events/total)	Control (events/total)	Statistical method	Effect estimate
Verstovsek *et al,* 2012	17/155	16/151	OR (M–H, Fixed, 95% CI)	1.04 (0.50, 2.14)	-	-	-	-
Harrison *et al,* 2012	12/146	4/73	OR (M–H, Fixed, 95% CI)	1.54 (0.48, 4.97)	92/146	11/73	OR (M–H, Fixed, 95% CI)	9.60 (4.66, 19.81)

OR, odds ratio; M-H, Mantel-Haenszel; CI, confidence interval.
